# Predicting biochemical-recurrence-free survival using a three-metabolic-gene risk score model in prostate cancer patients

**DOI:** 10.1186/s12885-022-09331-8

**Published:** 2022-03-04

**Authors:** Yiqiao Zhao, Zijia Tao, Lei Li, Jianyi Zheng, Xiaonan Chen

**Affiliations:** Department of Urology, Shengjing Hospital of China Medical University, Shenyang, Liaoning 110004 People’s Republic of China

**Keywords:** Prostate cancer, TCGA, Bioinformatics, Risk model, Metabolic studies, Gene set enrichment analysis

## Abstract

**Background:**

Biochemical recurrence (BCR) after initial treatment, such as radical prostatectomy, is the most frequently adopted prognostic factor for patients who suffer from prostate cancer (PCa). In this study, we aimed to construct a prognostic model consisting of gene expression profiles to predict BCR-free survival.

**Methods:**

We analyzed 70 metabolic pathways in 152 normal prostate samples and 494 PCa samples from the UCSC Xena dataset (training set) via gene set enrichment analysis (GSEA) to select BCR-related genes and constructed a BCR-related gene risk score (RS) model. We tested the power of our model using Kaplan–Meier (K–M) plots and receiver operator characteristic (ROC) curves. We performed univariate and multivariate analyses of RS using other clinicopathological features and established a nomogram model, which has stronger prediction ability. We used GSE70770 and DFKZ 2018 datasets to validate the results. Finally, we performed differential expression and quantitative real-time polymerase chain reaction analyses of the UCSC data for further verification of the findings.

**Results:**

A total of 194 core enriched genes were obtained through GSEA, among which 16 BCR-related genes were selected and a three-gene RS model based on the expression levels of CA14, LRAT, and MGAT5B was constructed. The outcomes of the K–M plots and ROC curves verified the accuracy of the RS model. We identified the Gleason score, pathologic T stage, and RS model as independent predictors through univariate and multivariate Cox analyses and constructed a nomogram model that presented better predictability than the RS model. The outcomes of the validation set were consistent with those of the training set. Finally, the results of differential expression analyses support the effectiveness of our model.

**Conclusion:**

We constructed an RS model based on metabolic genes that could predict the prognosis of PCa patients. The model can be easily used in clinical applications and provide important insights into future research on the underlying mechanism of PCa.

**Supplementary Information:**

The online version contains supplementary material available at 10.1186/s12885-022-09331-8.

## Introduction

In the past five years, the incidence and mortality rates of prostate cancer (PCa) in most regions worldwide have stabilized or decreased [1]; however, it is still the most common cancer in men worldwide [2]. Although a variety of curative treatments are available for PCa patients, such as radical prostatectomy (RP) or radiotherapy (RT) [3–8], patients showed approximately 20–40% and 30–50% biochemical recurrence (BCR) rates within approximately 10 years after receiving RP and RT, respectively [9–11]. BCR, which is defined as an increase in the blood level of prostate-specific antigen, indicates that the cancer has come back. Absence of specific guidelines for doctors to treat BCR [12] necessitates the identification of novel indicators of BCR to develop prognostic and therapeutic strategies for patients with PCa.

Bioinformatics methods are currently widely adopted in cancer research, and it is common for researchers to use a myriad of genes to screen differentially expressed genes at both ends (dramatically upregulated and downregulated genes) from an elaborately chosen gene list [13–16]. However, a drastic elevation in the expression of a single gene may exert less impact on the flux through the metabolic pathway than a small increase in the expression of all genes participating in the pathway. To address this limitation, a gene set enrichment analysis (GSEA) tool was developed to directly assess microarray data at the gene set level [17].

Gene sets usually originate from biochemical pathways that are frequently correlated with multiple cancers [16, 18–20]. PCa is also associated with metabolic pathways [21–27]; e.g., amplification of the Rac pathway and nicotinamide adenine dinucleotide metabolites has been identified as a boost for tumorigenesis in PCa [10]. Abnormalities in citrate and choline metabolism that occur in PCa samples were previously studied, and four component genes (ACLY, ACON, PLA2G7, and CHKA) of this metabolic pathway were identified as potential therapeutic strategies [9]. PCa has also been studied using metabolic genes in the past few years. For instance, CYP3A4 and CYP17 were found to be associated with PCa in African-American patients [28, 29], and androgen receptor and other androgen metabolic genes were found to be related to the progression of PCa; however, few researchers have constructed metabolic gene models for predicting the progression and prognosis of PCa patients.

**Fig. 1:**
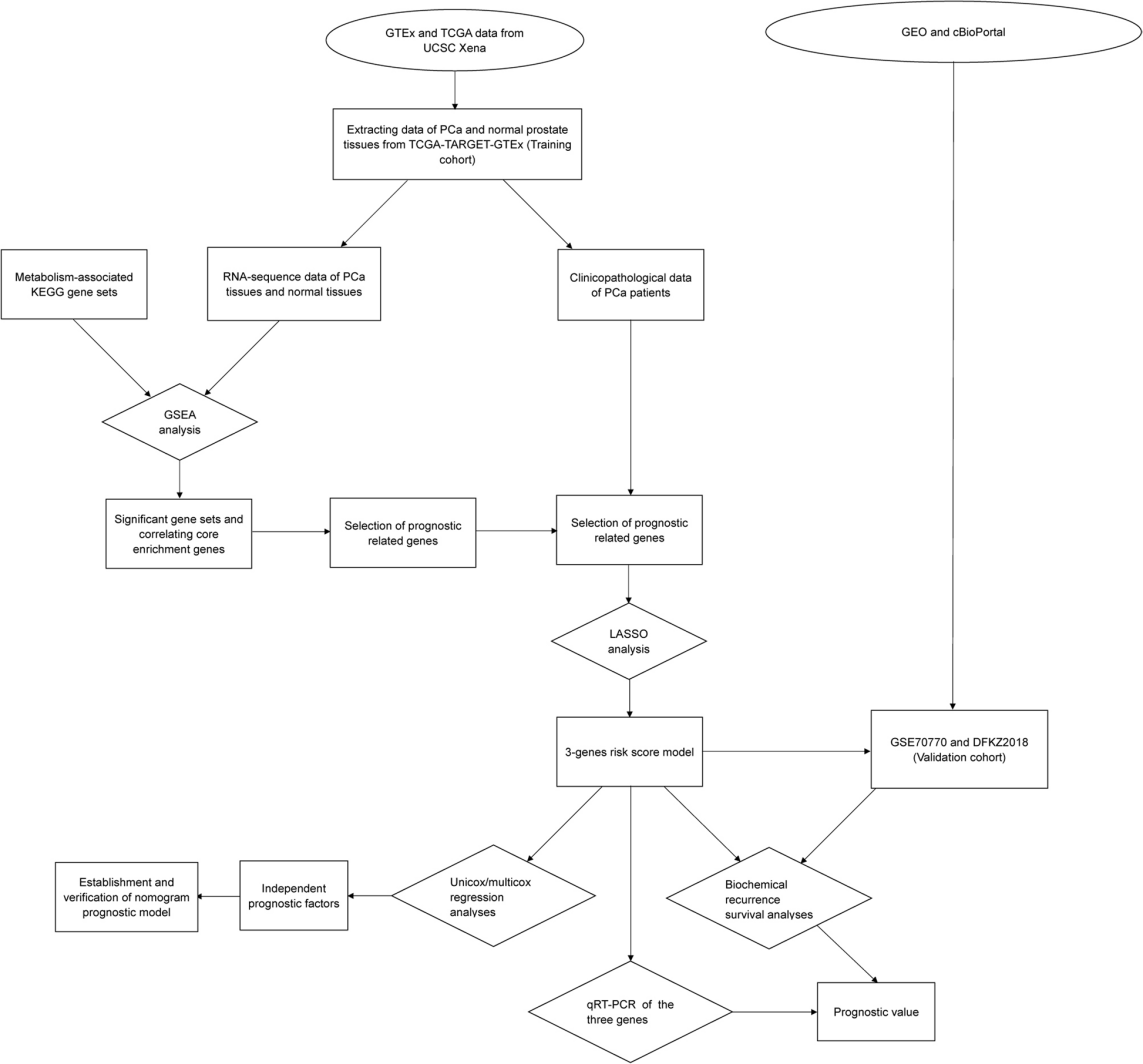
Overall work flow of this study. *GTEx:* Genotype-Tissue Expression project; TCGA: The Cancer Genome Atlas; GEO: Gene Expression Omnibus; PCa: prostate cancer; KEGG: Kyoto Encyclopedia of Genes and Genomes; GSEA: Gene Sets Enrichment Analysis; LASSO: Least absolute shrinkage and selection operator; qRT–PCR: quantitative real-time PCR

This study aims to construct a metabolic gene risk score (RS) model to predict PCa progression based on the expression levels of metabolic genes from The Cancer Genome Atlas (TCGA) database (including 494 PCa samples and 52 benign prostate samples) and the Genotype-Tissue Expression (*GTEx*) project (consisting of 100 benign prostate samples). The tools developed in this study will provide novel insights into the underlying mechanism of PCa at the molecular level. The workflow of this study is illustrated in Fig. 1.

## Materials and Methods

### Data Preparation

Gene expression information (the data type was RNA-Seq by expectation–maximization transcripts per kilobase million [RSEM TPM], including 152 normal samples and 494 tumor samples), and high-throughput sequencing fragments per kilobase million (HTSeq-FPKM), of 551 samples were collected from datasets “TCGA TARGET GTEx” and “GDC TCGA Prostate Cancer (TCGA-PRAD),” respectively, which are available at the University of California Santa Cruz (UCSC) Xena database (http://xena.ucsc.edu/). In addition, the clinical data of PCa patients were downloaded from the TCGA (https://portal.gdc.cancer.gov/). Patients with missing biochemical recurrence (BCR) data (including time to BCR and BCR state data) were excluded in this study. A total of 458 PCa patients who had both unabridged BCR data and gene expression data were incorporated for survival analysis. Moreover, among these 458 patients, 451 with complete clinicopathological data (gleason score, age and pathologic T stage) were utilized for the construction of risk score model in further analyses.

We obtained 70 Kyoto Encyclopedia of Genes and Genomes (KEGG) metabolism pathways as the source of metabolic genes [16, 30–32]. Two validation cohorts, GSE70770 (*n* = 203) and DFKZ 2018 (*n* = 82), were obtained from the Gene Expression Omnibus (GEO) (https://www.ncbi.nlm.nih.gov/geo/) and cBioPortal (https://www.cbioportal.org/), respectively. The clinical information of the training and validation cohorts is shown in Additional files 1 and 2, and 70 pathways and related genes are shown in Additional file 3.

### Metabolic genes related to biochemical recurrence

We analyzed the gene expression profiles (transformed from FPKM to TPM using R software, version 4.0.3) of the 70 KEGG pathways using GSEA (version 4.1.0). We extracted core enrichment genes (CEGs) from pathways with nominal p value < 0.05 and false discovery rate (FDR) < 0.25 and used them for univariate Cox regression analysis of the data from 458 patients with BCR information. Genes with p < 0.05 were regarded as BCR-related genes.

### RS model establishment

The RS model was constructed using the least absolute shrinkage and selection operator (LASSO) method.

RS = ∑n i = 1 each gene’s expression level*relevant coefficient.

A Kaplan–Meier plot (K–M plot) was mapped to compare the BFS rates. Furthermore, three- and five-year time-dependent receiver operating characteristic (ROC) curves of the RS were employed to assess predictability.

### Nomogram model construction and validation

We applied univariate and multivariate Cox regression analyses for independent predictors, and established a nomogram model using independent predictors for better prediction. We mapped ROC and calibration curves to assess the predictive efficiency of the model and established three- and five-year decision curve analyses of the nomogram model. These analyses can assist PCa patients’ decision-making on whether or not to receive further treatments [33].

### Validation of the RS model

We used two datasets (DFKZ 2018 and GSE70770), as described in the “Data preparation” section, to validate the RS model. We transformed the data into the TPM format to ensure consistency with the training dataset. We applied the RS model to the validation datasets and divided them into two groups according to their respective medians. We then performed corresponding survival and ROC analyses for comparison with the results of the training cohort.

We also constructed a violin plot to visualize the expression of the RS component genes in normal prostate/PCa tissues from the UCSC data. We detected the expression of these genes in 15 pairs of PCa and matched adjacent normal prostate tissues by performing quantitative real-time polymerase chain reaction (qRT–PCR) analysis (Additional file 4).

According to the product protocol, total RNA was extracted using TRIzol (Invitrogen, Waltham, MA, USA), and cDNA was synthesized via reverse transcription using the Transcript First‐Strand cDNA Synthesis Supermix Kit (Transgen Biotech, Beijing, China). SYBR premix Ex Taq II (Takara, Dalian, China) was used to detect the relative expression of the genes included in the model using qRT–PCR, and GAPDH was used as the internal reference. All reactions were repeated three times. Relative expression levels of these genes were calculated using the 2^−ΔΔCT^ method.

#### Statistical Analysis

All statistical analyses were performed using R software (version 4.0.3). We used t-test to analyze PCR data. The objective, method, and package name of all R packages used in this study are presented in Additional file 5.

## Results

### Acquiring CEGs

With the cutoff criteria mentioned in the “Metabolic genes related to biochemical recurrence” section, among 70 KEGG pathways, we detected 11 pathways significantly enriched in normal tissues, as well as 2 pathways enriched in PCa tissues (Additional file 6). We extracted 194 CEGs that functioned in these pathways. The pathways and corresponding CEGs are presented in Additional file 7.

### Selection of BCR-related genes

Among 194 CEGs, 55 genes had a p-value < 0.05, as determined by univariate Cox regression analysis. Among these 55 genes, only 16 genes demonstrated the same trend as the GSEA results and univariate Cox regression results (e.g., retinol acyltransferase (LRAT) was found to be enriched in the normal group, indicating that it should be a protective gene rather than an oncogene, and its hazard ratio was < 1; therefore, it was selected as a BCR-related gene). Therefore, these 16 genes were included in the subsequent analyses (Additional file 8).

### Building and verifying the RS model

**Fig. 2:**
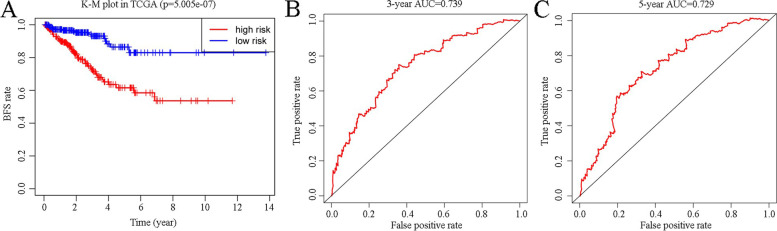
Evaluation of the RS model using Kaplan–Meier plot of the training set (divided into high- and low-risk groups based on the median of the risk score) (**A**), three-year BFS ROC curve (**B**), and five-year BFS ROC curve (**C**) predicted using the risk score model (*p* < 0.05 and AUC > 0.7). K–M plot, Kaplan–Meier plot; TCGA, The Cancer Genome Atlas; AUC, area under the curve; BFS, biochemical recurrence-free survival; ROC, receiver operating characteristic; RS, risk score

Of the 16 genes identified, 3 were screened via LASSO analysis, and using the expression level of these 3 genes, the RS model was formulated as (-0.0282084945656616)*(CA14 expression) + (-0.0475765886437412)*(LRAT expression) + 0.0419407402502097*(MGAT5B expression), where CA14 is carbonic anhydrase and MGAT5B is alpha-1,6-mannosylglycoprotein 6-beta-N acetylglucosaminyltransferase B. Patients were divided into two groups depending on the median RS, and then a K–M plot was constructed to compare the BCR of these two groups (*p* < 0.05) (Fig. 2A). The AUCs (areas under the curve**)** of three- and five-year ROC curves were 0.739 and 0.729 (Fig. 2B, C), respectively; both values being > 0.7 validates the accuracy of the RS model.

### Establishment of nomogram model

**Fig. 3:**
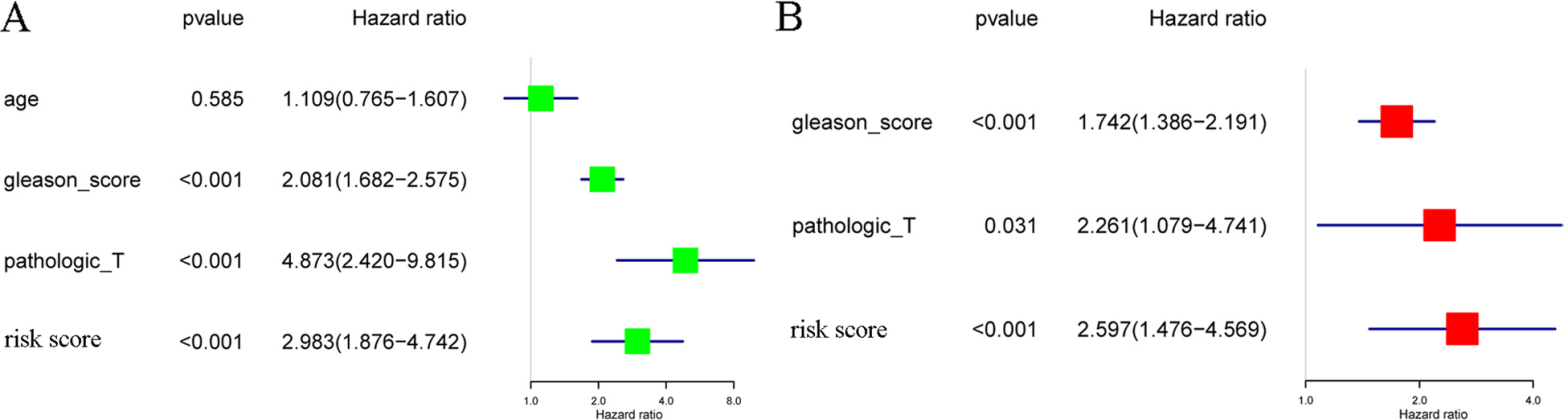
Univariate Cox analysis predicting prognostic factors (**A**) and multivariate Cox regression analysis identifying independent prognostic factors (**B**) among the RS model and other clinicopathological factors. In the “risk score” row, p value < 0.05 and hazard ratio > 1, indicating that the risk score model is a prognostic factor for PCa patients. AUC, area under the curve; RS, risk score; PCa, prostate cancer

**Fig. 4:**
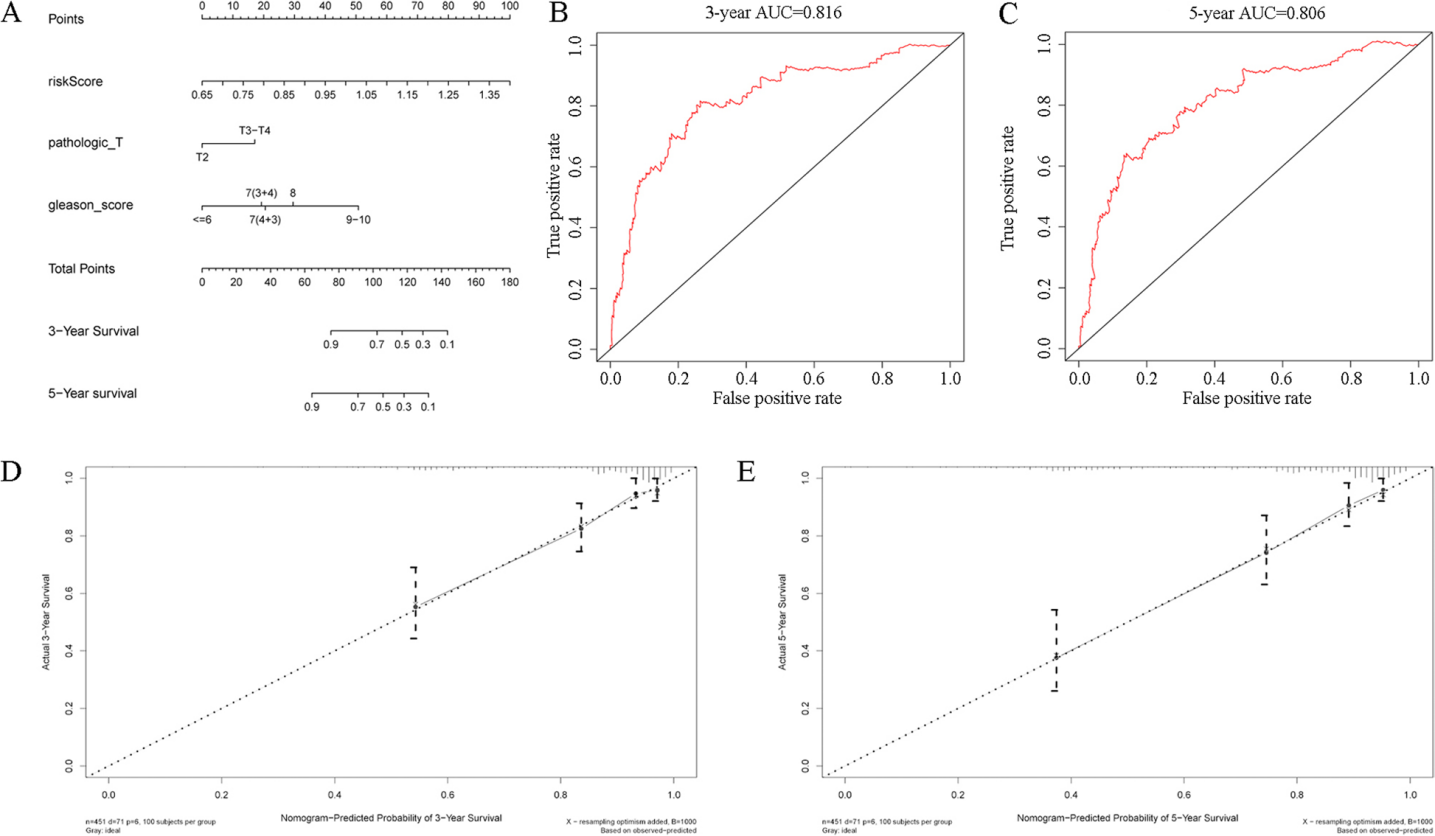
Establishment of the nomogram model using independent prognostic factors as a new prognostic model (**A**), three-year ROC curve and calibration curve of the model (**B**, **D**), and five-year ROC curve and calibration curve of the model (**C**, **E**). The results of the calibration and AUC were > 0.7, and the calibration curves showed good linearity for the three- and five-year BFS, revealing the predictive efficiency of the nomogram model. ROC, receiver operator characteristic; AUC, area under the curve; BFS, biochemical recurrence-free survival

**Fig. 5:**
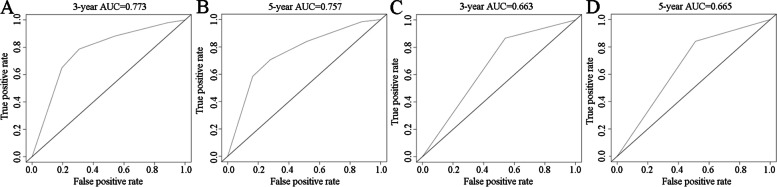
Three-year ROC curves of Gleason score and pathologic T stage (**A**, **C**) and five-year ROC curves of Gleason score and pathologic T stage for predicting the biochemical recurrence-free survival of PCa patients. The AUCs of T stage and Gleason score were lower than those of the nomogram model. AUC, area under the curve; ROC, receiver operating characteristic; PCa, prostate cancer

**Fig. 6:**
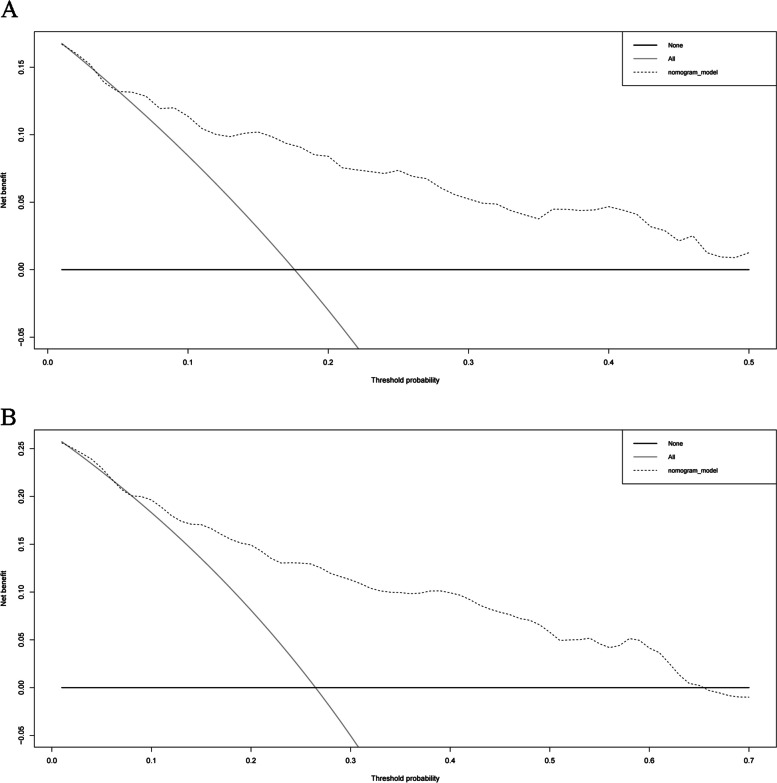
Decision curve analysis (DCA) of the nomogram model. The model has a high net benefit and a wide range of threshold probabilities in predicting the risk of biochemical recurrence within five (**A**) and three years (**B**). “None” means that no individual receives treatment or intervention. “All” means that all patients are treated or intervened clinically. The DCA presents a reference for patients who make decisions according to their respective anticipations

The Gleason score, pathologic T stage, and RS can be regarded as independent predictors according to the results of univariate and multivariate Cox regression analyses (based on the cutoff standard of p < 0.05) (Fig. 3A,B). A nomogram composed of the Gleason score, pathological T stage, and RS is shown in Fig. 4A. The corresponding AUCs were 0.816 and 0.806, respectively, which were larger than 0.7 (Fig. 4B, C), revealing the strong predictive capability of the nomogram model. The correlated calibration curves verified the capability of our model to predict BFS (Fig. 4D,E). We also calculated the AUCs of three- and five-year ROC curves for the Gleason score and pathologic T stage (Fig. 5A–D). The nomogram model was superior to all three independent predictors. We constructed a nomogram model with only T stage and Gleason score, calculated the AUCs of three- and five-year ROCs (Additional file 9), and observed that the AUCs (0.782 and 0.771, respectively) were lower than those of the nomogram model with RS (0.816 and 0.806, respectively). Moreover, we performed decision curve analysis based on the three- and five-year BFS of the nomogram model to support patient decision-making (Fig. 6A, B).

### Validation of the RS

**Fig. 7:**
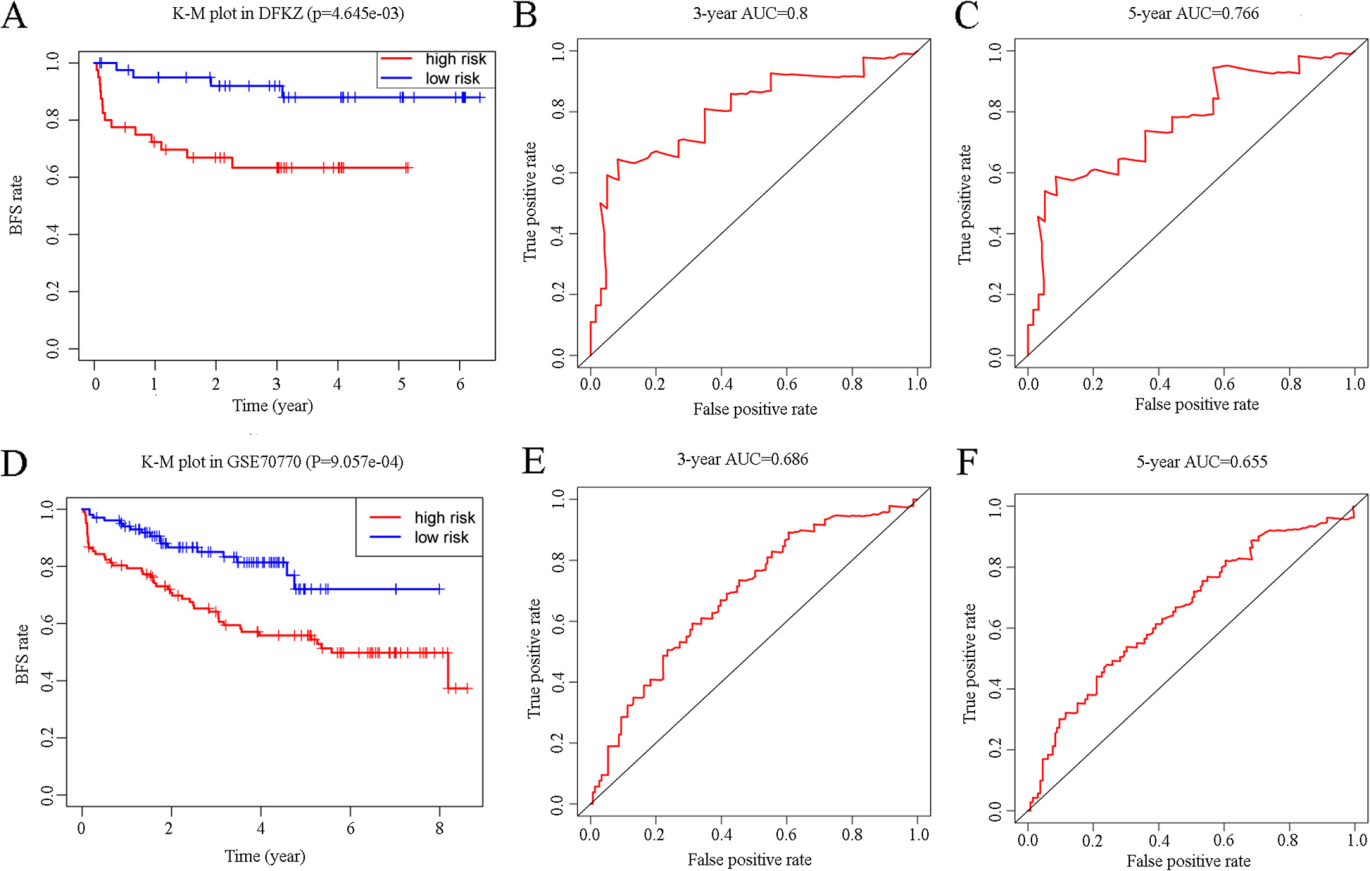
Kaplan–Meier curves of validation datasets DFKZ 2018 (**A**) and GSE70770 (**D**). The areas under the three- and five-year ROC curves of DFKZ 2018 (**B**, **C**) and GSE70770 (**E**, **F**) presented the same trend as the training set. K–M plot: Kaplan–Meier plot; AUC: area under curve

**Fig. 8:**
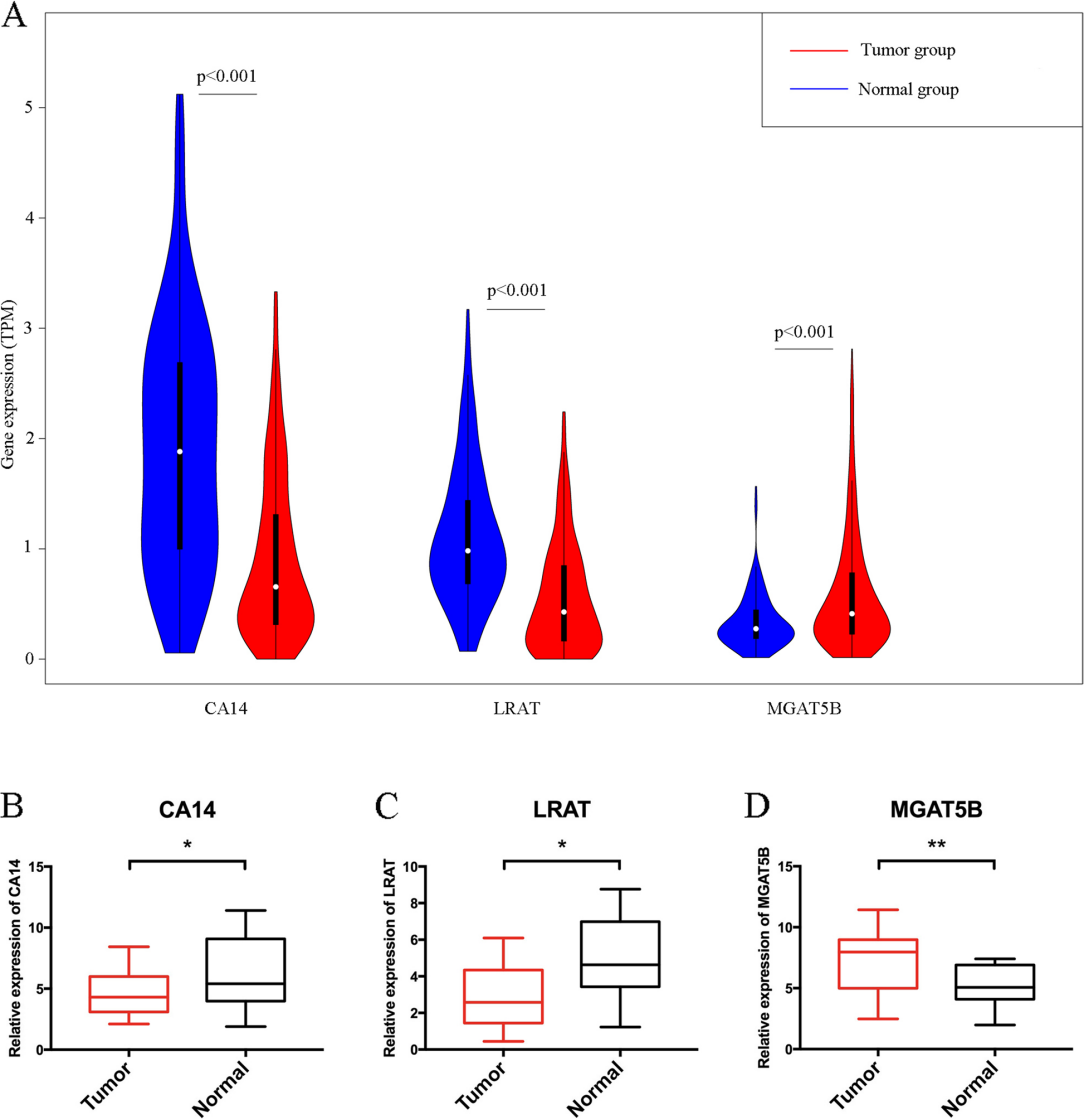
Comparison of the expression levels of CA14, LRAT, and MGAT5B in normal and malignant prostate tissues (**A**) using data from UCSC and using PCR (**B–D**). These three genes were differentially expressed in prostate cancer tissues. ‘*: *p* < 0.05; **: *p* < 0.01

Two validation cohorts from cBioPortal (DFKZ 2018) and GEO (GSE70770) were analyzed to further evaluate our risk score model. The corresponding K–M plots were constructed, and similar to the TCGA cohort, the p values of the K–M plots of both datasets were < 0.05 (Fig. 7A,D). The respective AUCs of three- and five-year DFKZ 2018 cohorts were 0.800 and 0.766 (Fig. 7B,C), while those of GSE70770 were 0.686 and 0.655 (Fig. 7E, F). We compared the differences in the expressions of RS component genes between normal prostate tissue and PCa tissues based on the TCGA data using the violin plot (Fig. 8A, *p* < 0.05 indicates statistical significance), which displays that expression levels of CA14 and LRAT were higher in normal tissues and that of MGAT5B was higher in tumor tissues. The corresponding qRT–PCR results of these three genes showed the same trend (Fig. 8B–D).

## Discussion

BCR can occur in > 65% of high-risk PCa patients after surgery [34–36]. Once BCR occurs, individuals are often recommended to receive subsequent treatment, even though BCR may not be a surrogate for progression or metastasis [12, 37]. Therefore, it is important to understand the molecular mechanisms of BCR.

In the present study, we filtered 194 metabolic genes via GSEA, selected 55 BFS-related genes from the TCGA (training set), and eventually established a 3-gene risk score. We then combined the risk score with other clinical features to generate a new model that could better predict BFS. Next, we validated the risk scores obtained from GSE70770 and DFKZ 2018 datasets (two validation sets). Finally, we used the PCR method to verify the results. All the results indicate that the risk score has strong predictive ability.

This is not the first study of BCR, which uses gene expression signatures [38–43]. In a previous study, a risk score model was constructed for a ferroptosis-related gene signature; the AUCs of three- and five-year ROC curves of the model in the TCGA cohort were 0.738 and 0.752 (close to the values obtained in our study, 0.739 and 0.729); however, most of these signatures contain too many genes that are not applicable to clinical tests. Those studies established a risk score composed of nine genes, which is three times the number of components in our signature, and evaluation using that risk score may cost more to patients/researchers for further treatment/research; therefore, it is less likely to be used in clinical applications. In addition, all genes selected in those studies were differentially expressed genes (DEGs). In the current study, we used GSEA to select candidate genes rather than DEGs, which focused more on the function of gene sets than on the expression level of a single gene so that genes functioning in tumors can be studied. Although our RS model is not excellent at the statistical level, it should still be further studied as a biomarker because it is based on biochemical metabolic pathways.

Our RS model consists of lecithin, LRAT, CA14, and MGAT5B. LRAT is a crucial component of retinol metabolism. According to data from cell lines provided by Guo et al. [44], the expression level of LRAT in PCa lines is significantly low. CA14, which was found to be expressed at higher levels in normal tissue than in tumor tissue, participates in nitrogen metabolism and is included in a gene signature designed to predict disease-free survival [45]. MGAT5B, which is involved in N-glycan biosynthesis, has been reported to be involved in metastasis competence in mice and is highly expressed in human PCa tissues [46]. Studies on PCa and metabolic pathways in which three constituent genes are involved have reported that gene expressions and gene variants in retinol metabolism are related to PCa prognosis [47–49]. Additionally, nitrogen metabolism has been found to correlate with PCa [50]. Serum N-glycan profiling has been identified as a potential biomarker for predicting prostate cancer prognosis [51, 52]. These past results are in line with those of our study and thus verify our results.

This study has some limitations. First, the cutoff values in the training and validation sets were selected based on the median value of the RS. Although we normalized the expression to TPM, the values still differed from each other. In addition, the data used in this study were from public datasets; therefore, further in vitro and in vivo experiments are necessary to support our findings. Furthermore, the training set was based on the PCa data of patients who live in the United States; therefore, the results and equations may not represent patients in other countries.

## Conclusions

We developed a risk score model to improve the prediction of biochemical recurrence in prostate cancer patients using metabolic genes and metabolic pathways. The results are highly consistent with the results of previous studies. The model can help explore the underlying mechanism of biochemical recurrence and provide new perspectives for the treatment or prevention of prostate cancer progression.

## Supplementary Information

**Additional file 1. **Clinical information of patients in thetraining cohort (TCGA-PRAD).

**Additional file 2. **Clinical information of patients in thevalidation cohort (DFKZ 2018) (A) and those in thevalidation cohort (GSE70770) (B).

**Additional file 3.** 70 KEGG metabolismpathways analyzed in this study. KEGG: Kyoto Encyclopedia of Genes and Genomes**.**

Additional file 4. Relative expression levels of CA14 (A), LRAT (B), and MGAT5B (C) inprostate cancer samples and matched normal samples used for PCR analysis.

**Additional file 5: **Objectives, methodsand package namesof different Rpackages used in different steps in our analysis.

**Additional file 6.** GSEA identifying KEGG pathways enriched in normal prostate tissues (A–K) and prostate cancer tissues (L, M). Gene Set Enrichment Analysis: Gene Set Enrichment Analysis; KEGG: Kyoto Encyclopedia of Genes andGenomes.

**Additional file 7.** Metabolism pathwaysand their corresponding core enrichment genes significantly enriched in normalsamples (A) and tumor samples (B). KEGG: Kyoto Encyclopedia ofGenes and Genomes.

**Additional file 8.** A list of coreenrichment genes related to the prognosis of prostate cancer patients.Each gene included had a p value < 0.05. HR,hazard ratio.

**Additional file 9.** The 3- and 5- year ROCcurves of the nomogram model constructed only by pathologic T stage and gleasonscore, the AUCs were lower than the nomogram model with the RS model,suggesting that the addition of our RS model increases could betterpredict BCR.

**Additional file 10.** The raw data from TCGA asnew raw data and data related to biochemical recurrence of prostate cancerpatients. There are three sheets in this file, the firstsheet “TCGA raw data” was the data directly obtained from TCGA dataset. In thesecond sheet “BCR state and time” we included data types we used for study.After excluding patients with missing “A8_New_Event_Time” data,we obtained **464** patients in sheet “data of464 patients”. BCR: Biochemical recurrence.

**Additional file 11.** Supplementary material legends

## Data Availability

The data that support the findings of this study are available in The Cancer Genome Atlas (TCGA) Gene Expression Omnibus (GEO) and cBioPortal. These databases are public databases, and their websites were provided in the manuscript. The expression data of qRT-PCR are uploaded as Additional file 4. Due to the complexity of the data on biochemical recurrence, we showed the data used as clinical data in Additional file 10. The legends of the supplementary files have been provided in Additional file 11.

## Abbreviations

BCR: Biochemical Recurrence
PCa: Prostate Cancer
GSEA: Gene Set Enrichment Analysis
K–M: Kaplan–Meier
RS: Risk Score
ROC: Receiver Operator Characteristic
RP: Radical Prostatectomy
RT: Radiotherapy
TCGA: The Cancer Genome Atlas
BFS: Biochemical Recurrence-Free Survival
FDR: False Discovery Rate
CEGs: Core Enrichment Genes
LASSO: Least Absolute Shrinkage and Selection Operator
qRT–PCR: Quantitative Real-Time Polymerase Chain Reaction
KEGG: Kyoto Encyclopedia of Genes and Genomes
DEGs: Differentially expressed genes
