# Supplementary material for: Predicting biochemical-recurrence-free survival using a three-metabolic-gene risk score model in prostate cancer patients
Source: BMC Cancer. 2022 Mar 4;22:239. doi: 10.1186/s12885-022-09331-8 (PMC8896158; doi:10.1186/s12885-022-09331-8)
Supplement: Supplementary file 1 — Additional file 1. Clinical information of patients in thetraining cohort (TCGA-PRAD). [file 12885_2022_9331_MOESM1_ESM.docx]

Table S1: General characteristics of the patients involved in training cohort

| Characteristics | N（%） |
| --- | --- |
| Age （years） |  |
| <60 | 180 (39.9) |
| ≥60 | 271 (60.1) |
| Gender |  |
| Male | 322 (66.3) |
| Female | 164 (33.7) |
| Pathologic T stage |  |
| T1 | 0 (0) |
| T2 | 181 (40.1) |
| T3 | 263 (58.3) |
| T4 | 7 (1.6) |
| Gleason score |  |
| 6 | 43 (9.5) |
| 7 (3+4) | 136 (30.2) |
| 7 (4+3)  8  9  10 | 95 (21)  53 (11.8)  122 (27)  2 (0.5) |
