# Supplementary material for: Predicting biochemical-recurrence-free survival using a three-metabolic-gene risk score model in prostate cancer patients
Source: BMC Cancer. 2022 Mar 4;22:239. doi: 10.1186/s12885-022-09331-8 (PMC8896158; doi:10.1186/s12885-022-09331-8)
Supplement: Supplementary file 2 — Additional file 2. Clinical information of patients in thevalidation cohort (DFKZ 2018) (A) and those in thevalidation cohort (GSE70770) (B). [file 12885_2022_9331_MOESM2_ESM.docx]

Table S2A: General characteristics of the patients involved in validation cohort DFKZ 2018

| Characteristics | N（%） |
| --- | --- |
| Age （years） |  |
| <60 | 82 (100) |
| ≥60 | 0 (0) |
| Pathologic T stage |  |
| T1 | 0 (0) |
| T2 | 56 (68.3) |
| T3 | 23 (28.1) |
| T4 | 3 (3.6) |

Table S2B: General characteristics of the patients involved in validation cohort GSE70770

| Characteristics | N（%） |
| --- | --- |
| Age （years） |  |
| <60 | 43 (21.2) |
| ≥60  NA | 68 (33.5)  92 (45.3) |
| Pathologic T stage |  |
| T0  T1 | 1 (0.5)  0 (0) |
| T2 | 81 (39.9) |
| T3 | 118 (58.1) |
| T4  Tx | 1 (0.5)  2 (1) |
| Gleason score |  |
| 5 | 2 (1) |
| 6 | 35 (17.2) |
| 7 (3+4)  7 (4+3) | 100 (49.3)  40 (19.7) |
| 8 | 13 (6.4) |
| 9  10  unknown | 10 (4.9)  1 (0.5)  2 (1) |
