# Supplementary material for: Predicting biochemical-recurrence-free survival using a three-metabolic-gene risk score model in prostate cancer patients
Source: BMC Cancer. 2022 Mar 4;22:239. doi: 10.1186/s12885-022-09331-8 (PMC8896158; doi:10.1186/s12885-022-09331-8)
Supplement: Supplementary file 5 — Additional file 5: Objectives, methodsand package namesof different Rpackages used in different steps in our analysis. [file 12885_2022_9331_MOESM5_ESM.docx]

Table S5: Objectives, methods and package names of different R packages in our analysis.

| Objective | Method | Package name |
| --- | --- | --- |
| Transformation of FPKM data to TPM data | NA | ‘data.table’, ‘tibble’, ‘dplyr’, and ‘tidyr’ R packages |
| Select BFS-related metabolic genes | univariate Cox regression analysis | ‘survival’ R package |
| Construction of LASSO Cox model | Cox regression analysis | ‘survival’ and ‘glmnet’ R package |
| Differences in survival between high and low risk groups | Kaplan-Meier survival analysis | ‘survival’ R package |
| Predictive performance  in different times | Time-dependent receiver operating characteristic analysis | ‘survivalROC’ package |
| Selection of independent predictors | Univariate and multivariate Cox regression analysis | ‘survival’ and ‘forestplot’ R package |
| Establishment of the nomogram model of independent factors | Cox regression analysis | ‘survival’, ‘foreign’ and ‘rms’ R package |
| Calibration curves of the nomogram model | Cox regression analysis | ‘survival’, ‘foreign’ and ‘rms’ R package |
| Clinical usefulness | Decision curve analysis | stdca.R |
| Comparison of expression levels of the three component genes | Wilcoxon Signed Rank Test | ‘vioplot’ package |

Abbreviations: FPKM= Fragments Per Kilobase Million; TPM= transcripts per kilobase million; NA= not available; BFS= biochemical recurrence-free survival; LASSO= Least absolute shrinkage and selection operator; ROC= receiver operating characteristic
