# Supplementary figures and images for: Predicting biochemical-recurrence-free survival using a three-metabolic-gene risk score model in prostate cancer patients
Source: BMC Cancer. 2022 Mar 4;22:239. doi: 10.1186/s12885-022-09331-8 (PMC8896158; doi:10.1186/s12885-022-09331-8)

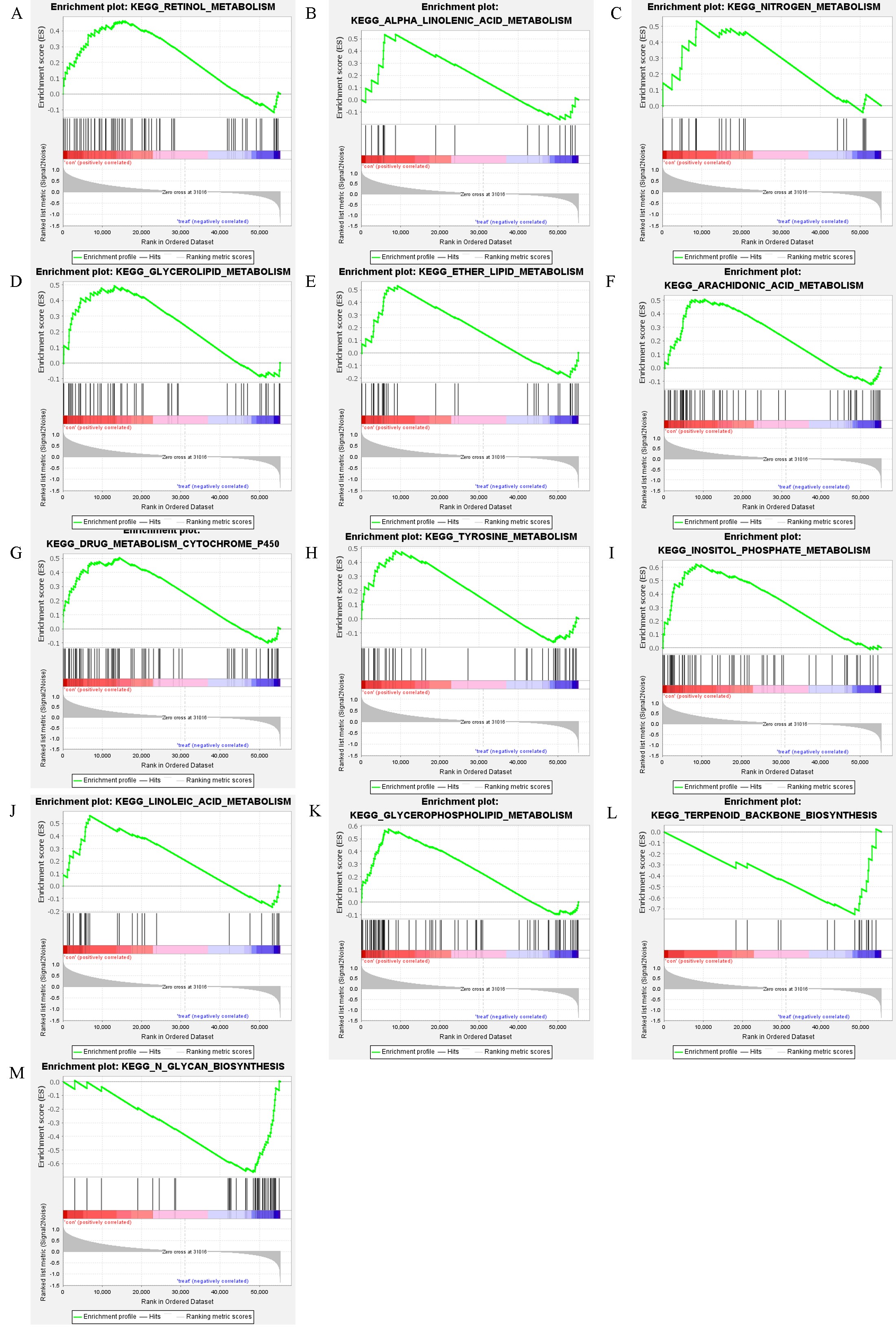

Supplement: Supplementary file 6 — Additional file 6. GSEA identifying KEGG pathways enriched in normal prostate tissues (A–K) and prostate cancer tissues (L, M). Gene Set Enrichment Analysis: Gene Set Enrichment Analysis; KEGG: Kyoto Encyclopedia of Genes andGenomes. [file 12885_2022_9331_MOESM6_ESM.jpg]

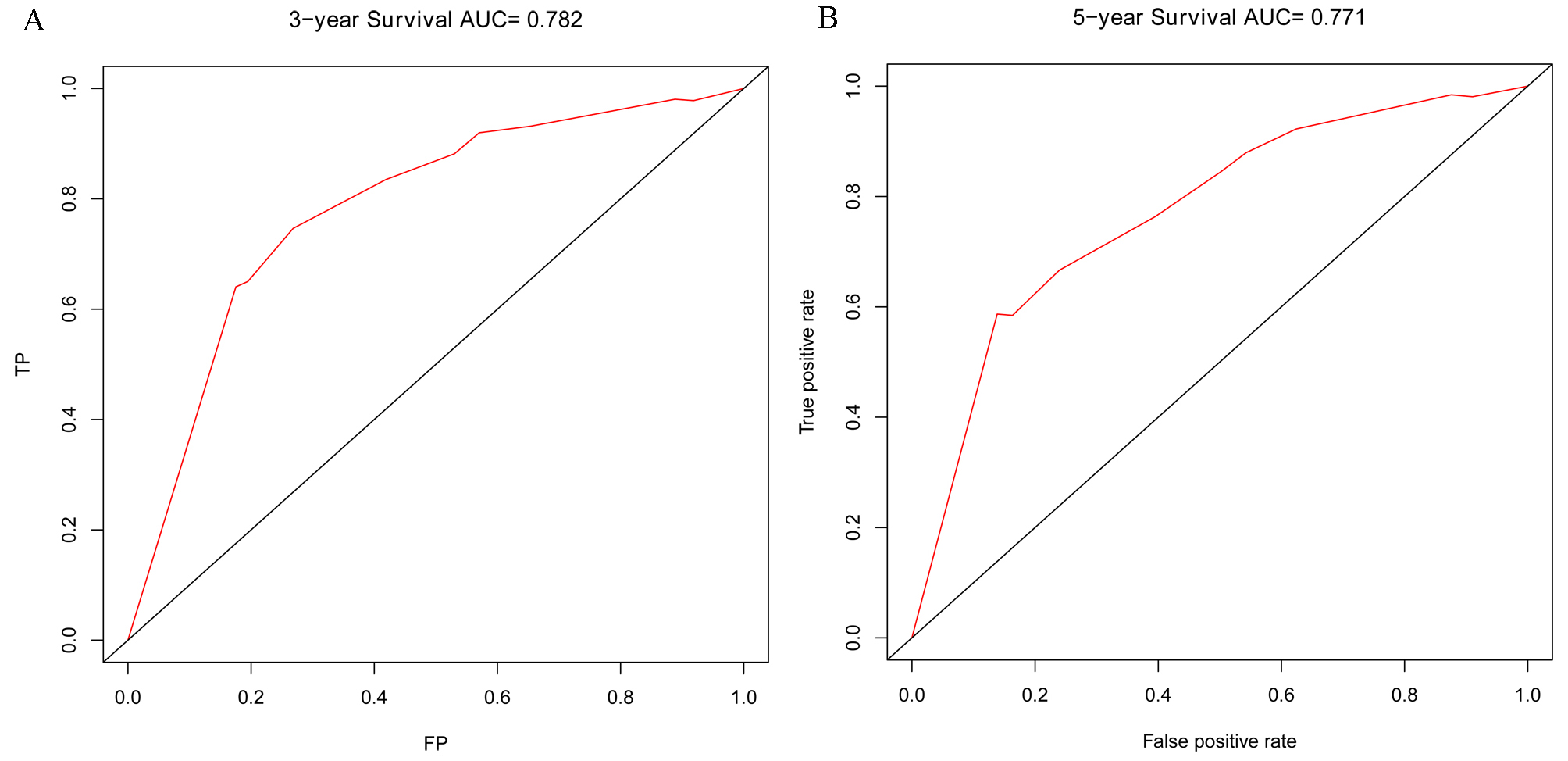

Supplement: Supplementary file 9 — Additional file 9. The 3- and 5- year ROCcurves of the nomogram model constructed only by pathologic T stage and gleasonscore, the AUCs were lower than the nomogram model with the RS model,suggesting that the addition of our RS model increases could betterpredict BCR. [file 12885_2022_9331_MOESM9_ESM.jpg]
