# Supplementary material for: Predicting biochemical-recurrence-free survival using a three-metabolic-gene risk score model in prostate cancer patients
Source: BMC Cancer. 2022 Mar 4;22:239. doi: 10.1186/s12885-022-09331-8 (PMC8896158; doi:10.1186/s12885-022-09331-8)
Supplement: Supplementary file 11 — Additional file 11. Supplementary material legends [file 12885_2022_9331_MOESM11_ESM.docx]

**Supplementary material legends**

Additional file 1: Clinical information of patients in the training cohort (TCGA-PRAD).

Additional file 2: Clinical information of patients in the validation cohort (DFKZ 2018) (A) and those in the validation cohort (GSE70770) (B)

Additional file 3: 70 KEGG metabolism pathways analyzed in this study. KEGG: Kyoto Encyclopedia of Genes and Genomes

Additional file 4: Relative expression levels of CA14 (A), LRAT (B), and MGAT5B (C) in prostate cancer samples and matched normal samples used for PCR analysis.

Additional file 5: Objectives, methods and package names of different R packages used in different steps in our analysis.

Additional file 6: GSEA identifying KEGG pathways enriched in normal prostate tissues (A–K) and prostate cancer tissues (L, M). Gene Set Enrichment Analysis: Gene Set Enrichment Analysis; KEGG: Kyoto Encyclopedia of Genes and Genomes.

Additional file 7: Metabolism pathways and their corresponding core enrichment genes significantly enriched in normal samples (A) and tumor samples (B). KEGG: Kyoto Encyclopedia of Genes and Genomes

Additional file 8: A list of core enrichment genes related to the prognosis of prostate cancer patients. Each gene included had a p value <0.05. HR, hazard ratio

Additional file 9: The 3- and 5- year ROC curves of the nomogram model constructed only by pathologic T stage and gleason score, the AUCs were lower than the nomogram model with the RS model, suggesting that the addition of our RS model increases could better predict BCR.

Additional file 10: The raw data from TCGA as new raw data and data related to biochemical recurrence of prostate cancer patients. There are three sheets in this file, the first sheet “TCGA raw data” was the data directly obtained from TCGA dataset. In the second sheet “BCR state and time” we included data types we used for study. After excluding patients with missing “A8_New_Event_Time” data, we obtained **464** patients in sheet “data of 464 patients”. BCR: Biochemical recurrence.
